# MIDDLE-AGED AND OLDER CAREGIVERS’ LONELINESS AND NEIGHBORHOOD DISORDER: THE BUFFERING ROLE OF SOCIAL SUPPORT

**DOI:** 10.1093/geroni/igae098.0826

**Published:** 2024-12-31

**Authors:** Seungjong Cho

**Affiliations:** Texas Tech University, Lubbock, Texas, United States

## Abstract

This presentation aims to explore the relationship between perceived neighborhood disorder and loneliness among middle-aged and older (age 50+) caregivers using the 2018 Health and Retirement Study (N = 578). It also identifies the role of social support as a buffer. The association between perceived neighborhood disorder, social support, and loneliness is an emerging area of research, especially for middle-aged and older caregivers. For statistical analysis, loneliness was measured as summed scores of 11 items asking how respondents felt about different aspects of their lives. Perceived neighborhood disorder was measured as summed scores of eight items of physical disorder and social disorder. Social support was measured as summed scores of 12 items asking about positive interactions with spouse/partner, children, relatives, and friends. A multiple regression model (Adjusted R-square =.296) showed that higher levels of perceived neighborhood disorder were associated with higher levels of loneliness (Beta =.222, p <.001). Social support had no significant buffering effect on this association (Beta = -.024, p =.495). However, social support itself was significantly associated with loneliness in an anticipated direction (Beta = -.400, p <.001). Among the covariates (gender, age, education, and self-rated health), only self-rated health was significantly related to loneliness (Beta = -.146, p <.001). This study highlights the importance of identifying and managing loneliness and neighborhood stressors among this unique population with the power of social support from their spouse/partner, children, relatives, and friends.

